# Dietary Fiber Analysis of Four Pulses Using AOAC 2011.25: Implications for Human Health

**DOI:** 10.3390/nu8120829

**Published:** 2016-12-21

**Authors:** Yiran Chen, Rebecca McGee, George Vandemark, Mark Brick, Henry J. Thompson

**Affiliations:** 1 Cancer Prevention Laboratory, Colorado State University, Fort Collins, CO 80523, USA; yiran.chen@colostate.edu; 2 Agricultural Research Service, United States Department of Agriculture, Washington State University, Pullman, WA 99164, USA; rebecca.mcgee@ars.usda.gov (R.M.); george.vandemark@ars.usda.gov (G.V.); 3 Department of Soil and Crop Sciences, Colorado State University, Fort Collins, CO 80523, USA; mark.brick@colostate.edu

**Keywords:** dietary fiber, pulse crops, dietary fiber gap, common bean, chickpeas, dry peas, lentils, AOAC 2011.25

## Abstract

Chickpeas, common beans, dry peas, and lentils are pulse crops that have been a cornerstone of the human diet since the inception of agriculture. However, the displacement of pulses from the diet by low fiber protein alternatives has resulted in a pervasive deficiency referred to as the dietary fiber gap. Using an analytical method American Association of Analytical Chemists (AOAC) 2011.25 that conforms to the Codex Alimentarius Commission consensus definition for dietary fiber, the fiber content of these pulse crops was evaluated in seed types used for commercial production. These pulse crops have 2 to 3 times more fiber per 100 g edible portion than other dietary staples. Moreover, there is marked variation in fiber content among cultivars of the same crop. We conclude that pulse crop consumption should be emphasized in efforts to close the dietary fiber gap. The substantial differences in fiber content among currently available cultivars within a crop can be used to further improve gains in fiber intake without the need to change dietary habits. This provides a rationale for cultivar-based food labeling.

## 1. Introduction

Dietary fiber is an important non-nutritive component of food, and is believed to have various benefits to human health [1]. In many countries, such as the United States and Canada, the intake of dietary fiber is 50% to 70% below recommended levels in greater than 95% of the population [2,3]. We recently advanced the concept that low dietary fiber intake could be partially accounted for by the infrequent or low consumption of pulse crops [4]. As defined by Food and Agriculture Organization (FAO) of the United Nations, pulses are grain legumes; the four most widely consumed pulse crops globally are chickpeas (*Cicer arietinum* L.), common beans (*Phaseolus vulgaris* L.), dry peas (*Pisum sativum* L.), and lentils (*Lens culinaris* L.) [5]. In many cultures, these pulse crops are food staples; i.e. they are eaten daily in large amounts and are both affordable and accessible to the population [6]. While this continues to be true in many developing countries, in other countries, such as the United States and Canada, pulse consumption has been displaced with animal products, oil seed legumes such as soy (protein isolate), and peanuts. Consequently, while the FAO recommends the consumption of cereal grains to pulses in a ratio of 2:1 (food servings), the current pattern of consumption globally is 8:1 [7]. Interestingly, it has been shown in both the United States and Canada that individuals whose consumption of pulses is high (highest quartile of dietary intake) meet recommended levels of dietary fiber intake in comparison to non-pulse consumers who eat the same daily number of servings of cereal grains [8,9].

While the first use of the term dietary fiber is attributed to Hipsley in 1953 [10], the hypothesis that dietary fiber is associated with human health benefits is attributed to the research team of Burkitt, Painter, Walker, and Trowel [11]. To support the conceptual development of their ideas, Trowel published a definition for dietary fiber in 1976 that was used for method development and policy formulation for over 20 years [12]. With the expansion of scientific knowledge, efforts to update Trowel’s definition have been ongoing [13]. Those efforts recently culminated in an international consensus by the authority responsible for determining global food standards and guidelines (the Codex Alimentarius Commission), with the adoption of a new definition of dietary fiber in 2009 by the Codex Committee on Nutrition and Foods for Special Dietary Uses [14]. The definition states that: “Dietary fiber consists of carbohydrate polymers with 10 or more monomeric units, which are not hydrolyzed by the endogenous enzymes in the small intestine of humans”. This definition resulted in a review of available analytical methods and whether the existing methods could define the fiber components included in the new definition. An integrated total dietary fiber analytical method was developed by McCleary et al. to comply with the 2009 CODEX definition and accurately measure total dietary fiber (TDF) as calculated by insoluble dietary fiber (IDF), soluble dietary fiber (SDF), and non-digestible oligosaccharides (OLIGO) content. This integrated method was subjected to inter-laboratory evaluations and initially accepted as AOAC 2009.01 [15]. Additional inter-laboratory evaluations resulted in the acceptance of AOAC method 2011.25 [16].

The consensus definition of dietary fiber and the acceptance of a method by which it can be reliably determined resolved a long-standing barrier to the field of dietary fiber and human health. We now apply AOAC 2011.25 as reported in [17] to the comparative analysis of pulse crops, since this information is not available in current nutrient databases. We also report on variability in dietary fiber among commercially-grown cultivars of each crop and demonstrate why efforts to brand foods with varietal information could help the consumer make more informed choices while providing an opportunity for food producers to advertise this value-added aspect of their product without the need to make any health claims.

## 2. Materials and Methods

### 2.1. Experimental Design 

Chickpea, common bean, dry pea, and lentil seed were selected by plant breeders familiar with commercial cultivars of each pulse crop grown in the United States. Chickpea was selected by Geroge Vandemark, common bean by Mark Brick, and dry peas and lentils were selected by Rebecca McGee. Seed was harvested from two locations, and within each location, two samples (replicates) of the same cultivar were obtained from different field plots.

### 2.2. Sample Preparation

Seed samples for analysis were treated as follows. Approximately 1 g of each seed sample was ground into a fine powder using a mechanical grinder (KRUPS Tipo203, Krups-USA, Peoria, IL, USA). Two subsamples of each sample were weighed and transferred into a 50-mL plastic Falcon tube (Corning Science Mexico, Reynosa, Mexico), and then 8 mL of Milli-Q water was added. The sample was vortexed with Baxter S/P vortex mixer (Baxter Diagnosis Inc., Deerfield, IL, USA) until the powder was well dispersed in water. The tubes were then placed in an autoclave, where they were autoclaved for 65 min at 115 °C with pressure of 76 kPa to simulate the cooking process. After cooking, the sample was cooled to room temperature and the contents of the tubes were fully homogenized using a Polytron^®^ PT10/35 (Kinematica, Lucerne, Switzerland) with an S type probe for approximately 15 s on speed 6. The probe was washed twice with 6 mL of Milli-Q water (EMD Millipore, Darmstadt, Germany) in a VWR Culture tube (VWR Scientific, West Chester, PA, USA) 17 × 100 mm after homogenization to remove all seed particles. The liquids from the two washings were transferred to the original 50 mL conical tube containing the homogenized seed sample. The final sample volume was approximately 20 mL. The conical tubes with the homogenized seed sample were stored at −80 °C until analysis.

### 2.3. AOAC 2011.25 Integrated Total Dietary Fiber Assay

Total integrated dietary fiber content was quantified per the AOAC 2011.25 method as modified by Kleintops et al. (2011) using a commercial assay kit (K-INTDF) purchased from Megazyme International, Wicklow, Ireland. The assay was carried out with modifications, as published in detail by our laboratory [17]. Briefly, the analysis of insoluble and soluble dietary fiber was per AOAC2011.25 without modification. Since oligosaccharides in pulses are limited to galactans, we used High Performance Liquid Chromatography (HPLC) separation with electrochemical detection to improve the sensitivity and specificity of the assay for raffinose, stachyose, and verbascose.

### 2.4. Statistical Analysis

An analysis of variance was carried out to compare entry means on all variables using the Procedure for General Linear Models (Proc GLM ) in SAS version 9.2 (SAS Institute Inc., Cary, NC, USA). Tukey’s multiple mean comparison method (*p* < 0.05) was used to determine significance among entry means and among crops for all variables.

## 3. Results

### 3.1. Chickpea 

Twenty-four kabuli chickpea entries were evaluated; they included five varieties and 19 breeding lines from the USDA-ARS Grain Legume Genetics and Physiology Research Unit (Pullman, WA, USA). Seed was obtained from field locations in Genesee, ID, and Pullman, WA. Selection of chickpeas was limited to the kabuli type, since that is the predominant type grown in the North America. Within each location, seed was collected from two different field plots. Since the goal of this study was to determine concentrations of dietary fiber components and total dietary fiber in cultivars of chickpea as they would be purchased and consumed, the four independent values for each variety were averaged and are reported in Table 1.

The average total dietary fiber for all chickpea cultivars was 21.8%, with a range of 19.5% to 24.9%. Average insoluble dietary fiber across the 24 cultivars was 15.8%, while the range was 14.4% to 17.1%. The average soluble dietary fiber for all cultivars was 3.5%, with a range of 2.0% to 5.8%. The average for total oligosaccharides was 2.5%, with a range between 1.0% and 3.5%.

### 3.2. Common Bean 

Twenty-six common bean entries were evaluated. Selection of seed included cultivars from market classes commonly grown in North America. Beans were obtained from two locations (Colorado and North Dakota), and within each location, seed was collected from two different field plots. Since the goal of this study was to determine concentrations of dietary fiber components and total dietary fiber in commercial cultivars of common bean as they would be purchased by a consumer, the four independent values for each variety were averaged and are reported in Table 2.

The average total dietary fiber for all common bean cultivars was 25.8%, with a range of 24.1% to 27.4%. The average IDF across cultivars was 13.9%, while the range was 12.3% to 15.7%. The average SDF for all cultivars was 7.7%, with a range of 5.7% to 9.8%. The average for total oligosaccharides was 4.2% with a range of 3.6% to 5.2%.

### 3.3. Dry Pea 

Eleven dry pea entries were evaluated, which included eight varieties and three breeding lines from the USDA-ARS Grain Legume Genetics and Physiology Research Unit (Pullman, WA, USA). Selection of dry peas cultivars were from market classes commonly grown in North America. Seed was obtained from two locations (Washington State and Idaho), and within each location, seed was collected from two different field plots (replicates). Since the goal of this study was to determine concentrations of dietary fiber components and total dietary fiber in varieties of dry pea as they would be purchased and consumed, the four independent values for each variety were averaged and are reported in Table 3.

The average total dietary fiber for all dry pea cultivars was 24.6%, with a range of 22.3% to 28.0%. The average for IDF across cultivars was 16.0%, while the range was 14.2% to 19.8%. The average SDF for all cultivars was 3.9%, with a range between 3.3% and 5.2%. The average for total oligosaccharides was 4.7%, with a range between 4.1% and 5.4%.

### 3.4. Lentils 

Thirteen lentil entries were evaluated, which included 11 varieties and two breeding lines. The selection of lentils included cultivars from market classes commonly grown in North America. Seed was obtained from two locations in Washington State—Pullman and Dayton—and within each location, seed was collected from two different field plots (replicates). Since the goal of this study was to determine concentrations of dietary fiber components and total dietary fiber in cultivars of lentils as they would be purchased and consumed, the four independent values for each variety were averaged, and are reported in Table 4.

The average total dietary fiber for lentil cultivars was 20.1% with a range from 18.4% to 21.3%. The average for IDF across cultivars was 13.6%, while the range was 12.2% to 14.7%. The average SDF for all cultivars was 3.2%, with a range between 2.7% and 3.9%. The average for total oligosaccharides was 3.3%, with a range between 3.0% and 3.7%.

### 3.5. Comparisons among Pulse Crops 

The data from Table 1, Table 2, Table 3 and Table 4 were compiled and then subjected to further statistical analyses. Table 5 summarizes the mean values across the pulse crops for total dietary fiber and its principle components, insoluble and soluble fiber, and for total oligosaccharides (i.e., raffinose, stachyose, and verbascose).

All pulse crops in this study had a high content of total dietary fiber. Common bean and dry pea have the highest amounts, which were statistically different from chickpea and lentil; however, when these data were reported in terms of TDF per serving as consumed or per 100 kcal as consumed, the relationships among pulse crops differed. Nonetheless, these differences are probably not clinically important, given the range in total dietary fiber across entries within each crop (Table 6), and the fact that identity preserved seed is currently not available to the consumer. Differences exist in dietary fiber components across pulse crops, and these are shown in Table 5.

## 4. Discussion

Dietary fiber influences human health, due in part to its effects on physiological functions, which include: (1) decreased intestinal transit time and increased stool bulk; (2) increased fermentation by colonic microflora; (3) reduced low-density lipoprotein cholesterol; and (4) reduced postprandial blood glucose and/or insulin [18,19]. These physiological effects have also been reported to impact the risk for several chronic diseases, including obesity, type 2 diabetes, heart disease, and certain types of cancer [20,21]. However, inadequate dietary fiber intake is widespread, with less than 10% of all Americans meeting recommended intake levels—a fact that has prompted renewed efforts to close the fiber gap in our diet [2,3,19].

The dietary fiber gap has existed for decades, despite strong efforts to increase fiber intake through consumer education and the introduction of an array of fiber enriched food products [22,23]. During the same period of time, an epidemic rate of obesity has occurred, a situation that strongly argues that foods with a high content of dietary fiber per 100 kcal portion should be emphasized in order to increase fiber intake without increasing caloric intake [24]. To this end, we have advanced evidence that the lack of pulse consumption in countries such as the United States is a significant contributor to the dietary fiber gap, since these crops provide two to three times more dietary fiber per 100 g edible portion than cereal grains, the type of foods frequently advertised as sources of dietary fiber [4]. The high protein and low fat content of pulses further strengthens the case for emphasizing their regular consumption in efforts to close the dietary fiber gap (Table 7).

Moreover, dietary surveys in both the United States and Canada demonstrate that pulse consumers in both countries exceed recommended levels of dietary fiber [8,9]. The magnitude of fiber intake in these pulse consumers is actually underestimated, since those studies used nutrient databases constructed before AOAC 2011.25 was published. Older methods reporting either crude fiber or acid detergent fiber and neutral detergent fiber values for pulse crops did not use enzymes in combination with chemicals for fiber extraction [25]. The crude fiber analysis isolates only a part of the insoluble fiber fraction, and greatly underestimates total dietary fiber content. Soluble fiber components are not measured in methods determining acid detergent fiber and neutral detergent fiber. The data in Table 1, Table 2, Table 3 and Table 4 resolve this issue, providing the fiber content of chickpeas, common bean, dry peas, and lentils using AOAC 2011.25. To our knowledge, this is the first reported application of this method to these four pulse crops.

Of the seventeen pulses recognized by FAO, the four crops investigated in this study are the most widely consumed pulses globally [5]. Of these, common bean is consumed in the largest amount. However, consumers who decide to increase pulse consumption to improve their dietary fiber status will undoubtedly ask whether one pulse is better than the others. Table 5 was constructed with this question in mind. While there were significant differences in dietary fiber components among pulse crops, with common bean and dry peas being the highest in overall fiber content, we consider that the best answer to the consumers’ question is to encourage consumption across pulse types, since it is recognized that consumption of a diverse array of dietary fiber components offers the approach most likely to facilitate tolerance of increasing levels of dietary fiber intake. Another common consumer question is whether specific components of dietary fiber should be emphasized (e.g., insoluble versus soluble dietary fiber). Historically, the answer to this question has varied, but the current consensus is that total fiber intake rather than consumption of a specific component is the most important consideration in terms of human health benefit [3,19]. With that in mind, a daily intake of 50 g dietary fiber, which was associated with a reduction in overall mortality in excess of 50% in the AARP cohort, might be considered a useful target intake for the health-oriented consumer [21].

A number of forces are driving a trend in consumer information and food labeling toward identity preservation of food sources, and in some cases, this extends to specific crop cultivars. Given that increasing dietary fiber intake is of paramount importance for human health and that there is no upper limit beyond which fiber intake is considered unsafe, Table 6 was constructed to summarize the amount of variation in dietary fiber components that exist among the cultivars of chickpea, common bean, dry pea and lentils that were evaluated [1]. The magnitude of these differences suggests that consideration should be given to cultivar labeling that underscores increased dietary fiber content in the absence of making any health claims. Choosing high versus low fiber cultivars of a particular pulse crop could boost total fiber intake by as much as 30%, increasing the feasibility of reaching or exceeding recommended levels of consumption in the absence of an increase in caloric intake.

There are a number of issues related to pulse consumption and dietary fiber intake that merit additional comment. Pulse seeds must be cooked prior to consumption to inactivate lectins and to make them palatable. If seed is soaked before it is cooked, and the water is discarded, the oligosaccharide content can be lower, and thus total dietary fiber consumed reduced. While oligosaccharide consumption is associated with flatulence, oligosaccharides are also known to exert prebiotic effects, and thus their consumption is thought to be beneficial. It is also known that cooking can affect physical characteristics of dietary carbohydrate, and that is why AOAC 2011.25 was applied to cooked seed as reported herein [26].

## 5. Conclusions

To close the fiber gap, pulse crops should be emphasized in the diet, since they have two to three times more fiber per 100 g than other dietary staples. Substantial differences in fiber content among cultivars within a crop allows for additional gains in fiber intake without the need to change dietary habits, and suggests a rationale for cultivar-based food labeling.

## Figures and Tables

**Table 1:** Dietary fiber content of chickpea cultivars and breeding lines.

Entry	IDF ^a,b^	SDF ^a,b^	OLIGO ^a,b^	TDF ^a,b^
Billy beans	15.4 ± 1.9 ^ABCD^	3.2 ± 0.3 ^EFG^	3.2 ± 0.1 ^AB^	21.8 ± 1.8 ^BCDEFG^
CA04900843C	16.9 ± 0.9 ^A^	3.4 ± 0.4 ^DEF^	3.1 ± 0.2 ^ABC^	23.4 ± 1.0 ^ABC^
CA0690B0427C	15.1 ± 1.2 ^ABCD^	5.8 ± 0.2 ^A^	1.9 ± 0.9 ^GH^	22.8 ± 1.7 ^ABCDEF^
CA0690B250C	16.7 ± 0.7 ^AB^	3.3 ± 0.2 ^DEFG^	3.1 ± 0.1 ^ABC^	23.2 ± 0.6 ^ABCD^
CA0790B0034C	16.5 ± 0.9 ^ABCD^	5.7 ± 0.6 ^A^	2.7 ± 0.6 ^ABCDEF^	24.9 ± 1.1 ^A^
CA0790B0042C	15.9 ± 1.0 ^ABCD^	5.8 ± 0.6 ^A^	2.8 ± 0.4 ^ABCDE^	24.5 ± 1.0 ^A^
CA0790B0043C	15.6 ± 1.1 ^ABCD^	2.0 ± 0.1 ^I^	3.3 ± 0.2 ^AB^	21.0 ± 1.2 ^DEFG^
CA0790B0053C	15.0 ± 2.6 ^ABCD^	2.0 ± 0.1 ^I^	3.0 ± 0.1 ^ABC^	19.9 ± 2.7 ^G^
CA0790B0054C	15.6 ± 1.2 ^ABCD^	2.4 ± 0.2 ^HI^	2.9 ± 0.2 ^ABCD^	20.9 ± 1.4 ^DEFG^
CA0790B0547C	15.6 ± 0.6 ^ABCD^	3.2 ± 0.3 ^EFG^	2.1 ± 0.7 ^DEFGH^	20.9 ± 0.7 ^EFG^
CA0790B0549C	15.8 ± 1.9 ^ABCD^	3.2 ± 0.6 ^EFG^	2.5 ± 0.9 ^BCDEFG^	21.4 ± 2.7 ^CDEFG^
CA0790B0642C	16.7 ± 1.1 ^AB^	2.8 ± 0.3 ^GH^	2.1 ± 0.6 ^EFGH^	21.5 ± 0.9 ^CDEFG^
CA0790B0733C	15.0 ± 1.2 ^ABCD^	3.0 ± 0.5 ^EFG^	1.8 ± 1.0 ^GH^	19.8 ± 2.0 ^G^
CA0890B0085W	14.4 ± 1.3 ^D^	3.3 ± 0.3 ^DEF^	1.9 ± 0.2 ^FGH^	19.6 ± 1.4 ^G^
CA0890B0429C	15.9 ± 0.9 ^ABCD^	2.9 ± 0.4 ^FGH^	2.1 ± 1.0 ^DEFGH^	20.9 ± 1.8 ^DEFG^
CA0890B0434C	16.0 ± 1.0 ^ABCD^	3.0 ± 0.6 ^FG^	2.4 ± 0.3 ^CDEFGH^	21.3 ± 1.2 ^CDEFG^
CA0890B0496C	14.7 ± 1.2 ^BCD^	3.0 ± 0.3 ^FG^	1.9 ± 1.1 ^FGH^	19.5 ± 2.2 ^G^
CA0890B0531C	16.2 ± 3.8 ^ABCD^	2.9 ± 0.2 ^FG^	1.6 ± 0.8 ^HI^	20.7 ± 3.4 ^FG^
CA0890B0551C	15.5 ± 1.6 ^ABCD^	3.8 ± 0.2 ^CD^	1.0 ± 0.9 ^I^	20.3 ± 1.9 ^G^
CA0890B0628W	14.6 ± 1.4 ^CD^	4.4 ± 0.5 ^B^	2.1 ± 1.2 ^DEFGH^	21.1 ± 2.0 ^DEFG^
CA0890B0648W	15.7 ± 1.2 ^ABCD^	4.3 ± 0.4 ^BC^	2.8 ± 0.1 ^ABCDE^	22.8 ± 1.4 ^ABCDEF^
CDC-Frontier	17.1 ± 1.9 ^A^	3.6 ± 0.2 ^DE^	3.2 ± 0.1 ^AB^	23.8 ± 1.9 ^AB^
Sawyer	16.6 ± 1.4 ^ABC^	3.4 ± 0.3 ^DEF^	3.4 ± 0.1 ^A^	23.4 ± 1.4 ^ABC^
Sierra	16.1 ± 1.7 ^ABCD^	3.5 ± 0.1 ^DEF^	3.5 ± 0.2 ^A^	23.1 ± 1.5 ^ABCDE^
Overall Mean	15.8 ± 1.5	3.5 ± 1.5	2.5 ± 0.9	21.8 ± 2.2
*p*-value	0.498	<0.0001	<0.0001	<0.0001

^a^ Values are expressed as percent of dry weight; means ± SD; ^b^ Means followed by different superscripted letters within a column are significantly different (*p* < 0.05) according to Tukey’s mean separation test. Abbreviations: IDF: insoluble dietary fiber; SDF: soluble dietary fiber; OLIGO: total oligosaccharides (raffinose + stachyose + verbascose); TDF: total dietary fiber (IDF + SDF + OLIGO).

**Table 2:** Dietary fiber content of common bean cultivars.

Variety	IDF ^a,b^	SDF ^a,b^	OLIGO ^a,b^	TDF ^a,b^
Agassiz	13.1 ± 0.5 ^DEF^	7.0 ± 0.2 ^EFGHI^	4.1 ± 0.2 ^BCDE^	24.1 ± 0.6 ^E^
Arthur	14.4 ± 0.5 ^ABCDE^	6.8 ± 0.4 ^FGHI^	3.6 ± 0.2 ^E^	24.8 ± 0.5 ^CDE^
Avalanche	14.3 ± 0.9 ^ABCDE^	6.6 ± 0.6 ^HI^	3.9 ± 0.4 ^CDE^	24.8 ± 0.9 ^DE^
Sequoia	13.4 ± 0.7 ^CDEF^	7.1 ± 0.1 ^EFGH^	4.3 ± 0.4 ^BCD^	24.9 ± 0.6 ^CDE^
Blackhawk	13.2 ± 0.3 ^CDEF^	7.1 ± 0.7 ^EFGH^	4.7 ± 0.2 ^AB^	25.1 ± 0.6 ^BCDE^
Fisher	13.8 ± 0.9 ^ABCDEF^	6.9 ± 0.3 ^EFGHI^	4.4 ± 0.4 ^BC^	25.2 ± 0.4 ^BCDE^
Buckskin	13.2 ± 0.7 ^DEF^	7.8 ± 0.5 ^CDEFGH^	4.3 ± 0.2 ^BCD^	25.3 ± 1.2 ^BCDE^
Beryl	12.3 ± 0.4 ^F^	9.2 ± 0.3 ^AB^	4.0 ± 0.2 ^CDE^	25.5 ± 0.3 ^BCDE^
Harrowhawk	13.8 ± 0.6 ^BCDEF^	7.6 ± 0.3 ^CDEFGH^	4.1 ± 0.1 ^BCDE^	25.5 ± 0.5 ^BCDE^
Hyden	13.6 ± 1.0 ^BCDEF^	8.1 ± 0.5 ^BCDEFG^	3.9 ± 0.2 ^CDE^	25.6 ± 1.1 ^ABCDE^
Bill Z	14.4 ± 1.0 ^ABCDE^	7.6 ± 0.5 ^CDEFGH^	3.6 ± 0.3 ^E^	25.6 ± 1.5 ^ABCDE^
Jaguar	13.7 ± 0.9 ^BCDEF^	7.8 ± 0.2 ^CDEFGH^	4.2 ± 0.2 ^BCDE^	25.7 ± 0.8 ^ABCDE^
Bunsi	14.1 ± 1.5 ^ABCDEF^	8.1 ± 0.8 ^BCDEFG^	3.7 ± 0.3 ^DE^	25.8 ± 0.9 ^ABCDE^
Gala	13.2 ± 0.4 ^CDFF^	8.1 ± 0.2 ^BCDEFG^	4.5 ± 0.4 ^ABC^	25.8 ± 1.0 ^ABCDE^
La Paz	14.6 ± 0.7 ^ABCD^	7.4 ± 0.5 ^DEFGH^	3.9 ± 0.5 ^CDE^	25.8 ± 1.3 ^ABCDE^
Pink Floyd	12.6 ± 0.3 ^EF^	9.1 ± 0.5 ^AB^	4.2 ± 0.4 ^BCDE^	26.0 ± 0.9 ^ABCDE^
Harold	12.9 ± 0.7 ^DEF^	8.8 ± 0.1 ^ABC^	4.4 ± 0.3 ^BC^	26.1 ± 1.0 ^ABCD^
Hatton	14.2 ± 1.0 ^ABCDE^	7.5 ± 1.2 ^DEFGH^	4.4 ± 0.3 ^BC^	26.1 ± 1.2 ^ABCD^
Aifi Wuriti	15.0 ± 0.7 ^ABC^	7.2 ± 0.3 ^EFGH^	4.1 ± 0.3 ^BCDE^	26.3 ± 0.9 ^ABCD^
Coulee	13.6 ± 1.2 ^BCDEF^	8.1 ± 0.2 ^BCDEF^	4.5 ± 0.3 ^BC^	26.3 ± 1.5 ^ABCD^
Garnet	14.0 ± 1.0 ^ABCDEF^	8.2 ± 0.5 ^BCDE^	4.2 ± 0.4 ^BCDE^	26.4 ± 1.1 ^ABCD^
Centa Pupil	15.7 ± 0.9 ^A^	5.7 ± 0.6 ^I^	5.2 ± 0.3 ^A^	26.6 ± 0.6 ^ABCD^
Frontier	13.9 ± 0.3 ^ABCDEF^	8.5 ± 0.5 ^BCD^	4.2 ± 0.3 ^BCDE^	26.7 ± 0.4 ^ABCD^
Rojo Chiquito	15.4 ± 1.4 ^AB^	6.8 ± 0.7 ^GHI^	4.5 ± 0.3 ^BC^	26.7 ± 1.0 ^ABC^
Jackpot	14.4 ± 2.0 ^ABCDE^	8.2 ± 1.0 ^BCDE^	4.4 ± 0.4 ^BC^	27.0 ± 1.4 ^AB^
Voyager	13.6 ± 0.8 ^BCDEF^	9.8 ± 0.7 ^A^	4.0 ± 0.3 ^CDE^	27.4 ± 0.8 ^A^
MEAN	13.9 ± 1.1	7.7 ± 1.0	4.2 ± 0.4	25.8 ± 1.1
*p*-value	0.0001	0.021	0.007	0.008

^a^ Values are expressed as percent of dry weight; means ± SD; ^b^ Means followed by different superscripted letters within a column are significantly different (*p* < 0.05) according to Tukey’s mean separation test. Abbreviations: IDF: insoluble dietary fiber; SDF: soluble dietary fiber; OLIGO: total oligosaccharides (raffinose + stachyose + verbascose); TDF: total dietary fiber (IDF + SDF + OLIGO).

**Table 3:** Dietary fiber content of dry pea cultivars and breeding lines.

Variety	IDF ^a,b^	SDF ^a,b^	OLIGO ^a,b^	TDF ^a,b^
HAMPTON	14.6 ± 1.4 ^C^	3.6 ± 0.7 ^B^	4.1 ± 0.9 ^DE^	22.3 ± 1.6 ^C^
PS03101445	14.2 ± 2.2 ^C^	3.5 ± 0.5 ^B^	5.2 ± 0.4 ^AB^	22.9 ± 2.1 ^C^
PS071019971	15.5 ± 1.4 ^BC^	3.8 ± 0.7 ^B^	4.4 ± 0.4 ^BCDE^	23.8 ± 1.7 ^C^
DS ADMIRAL	16.0 ± 1.2 ^BC^	3.5 ± 0.4 ^B^	4.3 ± 0.4 ^CDE^	23.8 ± 1.6 ^C^
COLUMBIAN	15.4 ± 0.5 ^BC^	3.3 ± 0.6 ^B^	5.4 ± 0.3 ^A^	24.1 ± 0.6 ^BC^
CAROUSEL	15.0 ± 1.4 ^C^	4.4 ± 0.6 ^AB^	4.8 ± 0.7 ^ABCD^	24.1 ± 2.3 ^BC^
PS08101022	15.8 ± 1.2 ^BC^	3.7 ± 1.5 ^B^	4.6 ± 0.3 ^ABCDE^	24.2 ± 0.8 ^BC^
ARAGORN	14.8 ± 1.7 ^C^	5.2 ± 2.0 ^A^	4.4 ± 0.5 ^CDE^	24.5 ± 1.1 ^BC^
SPECTER	17.6 ± 2.5 ^AB^	4.1 ± 0.5 ^AB^	4.9 ± 0.2 ^ABCD^	26.7 ± 3.0 ^AB^
MELROSE	19.8 ± 2.9 ^A^	3.9 ± 0.7 ^B^	4.0 ± 0.2 ^E^	27.6 ± 2.5 ^A^
GRANGER	19.1 ± 1.5 ^A^	3.8 ± 0.6 ^B^	5.0 ± 1.0 ^ABC^	28.0 ± 2.5 ^A^
Overall Mean	16.0 ± 2.2	3.9 ± 1.0	4.7 ± 0.6	24.6 ± 2.4
*p*-value	0.004	0.079	0.026	0.006

^a^ Values are expressed as percent of dry weight; means ± SD; ^b^ Means followed by different superscripted letters within a column are significantly different (*p* < 0.05) according to Tukey’s mean separation test. Abbreviations: IDF: insoluble dietary fiber; SDF: soluble dietary fiber; OLIGO: total oligosaccharides (raffinose + stachyose + verbascose); TDF: total dietary fiber (IDF + SDF + OLIGO).

**Table 4:** Dietary fiber content of lentil cultivars and breeding lines.

Variety	IDF ^a,b^	SDF ^a,b^	OLIGO ^a,b^	TDF ^a,b^
Cedar	12.2 ± 1.0 ^C^	3.1 ± 0.0 ^BCD^	3.0 ± 0.1 ^BC^	18.4 ± 1.1 ^F^
Morton	13.7 ± 0.8 ^B^	2.7 ± 0.1 ^E^	3.0 ± 0.0 ^C^	19.4 ± 0.7 ^E^
Pardina	13.5 ± 0.3 ^B^	2.8 ± 0.2 ^DE^	3.2 ± 0.1 ^ABC^	19.5 ± 0.1 ^DE^
LC08600113P	13.4 ± 0.3 ^B^	3.0 ± 0.1 ^BCDE^	3.4 ± 0.2 ^ABC^	19.8 ± 0.2 ^CDE^
CDC Viceroy	13.4 ± 0.3 ^B^	3.0 ± 0.1 ^BCDE^	3.4 ± 0.3 ^ABC^	19.8 ± 0.5 ^CDE^
Eston	13.5 ± 0.3 ^B^	3.0 ± 0.1 ^BCDE^	3.3 ± 0.3 ^ABC^	19.9 ± 0.2 ^CDE^
Richlea	13.7 ± 0.3 ^B^	3.2 ± 0.2 ^BC^	3.1 ± 0.2 ^BC^	20.0 ± 0.2 ^CD^
Avondale	13.6 ± 0.7 ^B^	3.1 ± 0.3 ^BCD^	3.3 ± 0.4 ^ABC^	20.0 ± 0.3 ^CD^
LC06601734L	13.7 ± 0.3 ^B^	3.3 ± 0.1 ^B^	3.3 ± 0.3 ^ABC^	20.3 ± 0.3 ^BC^
Merrit	13.5 ± 0.8 ^B^	3.3 ± 0.1 ^B^	3.5 ± 0.2 ^AB^	20.3 ± 0.8 ^BC^
Crimson	14.7 ± 0.4 ^A^	2.9 ± 0.0 ^CDE^	3.0 ± 0.3 ^C^	20.6 ± 0.7 ^B^
Brewer	13.8 ± 0.6 ^B^	3.3 ± 0.1 ^B^	3.7 ± 0.2 ^A^	20.8 ± 0.4 ^AB^
Pennell	13.8 ± 0.2 ^B^	3.9 ± 0.3 ^A^	3.6 ± 0.3 ^A^	21.3 ± 0.2 ^A^
Overall Mean	13.6 ± 0.6	3.2 ± 0.3	3.3 ± 0.3	20.1 ± 0.7
*p*-value	0.0155	<0.0001	0.1152	<0.0001

^a^ Values are expressed as percent of dry weight; means ± SD; ^b^ Means followed by different superscripted letters within a column are significantly different (*p* < 0.05) according to Tukey’s mean separation test. Abbreviations: IDF: insoluble dietary fiber; SDF: soluble dietary fiber; OLIGO: total oligosaccharides (raffinose + stachyose + verbascose); TDF: total dietary fiber (IDF + SDF + OLIGO).

**Table 5:** Dietary fiber content of pulse crops.

Crop	IDF ^a,b^	SDF ^a,b^	OLIGO ^a,b^	TDF ^a,b^	TDF ^c^ g/Serving	TDF ^d^ g/100 kcal
Chickpea	15.8 ± 0.8 ^A^	3.5 ± 1.0 ^A,C^	2.5 ± 0.7 ^C^	21.8 ± 1.6 ^B^	7.1	5.3
Common bean	13.9 ± 1.1 ^B^	7.7 ± 1.0 ^B^	4.2 ± 0.4 ^A^	25.8 ± 1.1 ^A^	8.1	6.6
Dry pea	16.2 ± 1.9 ^A^	3.9 ± 0.5 ^A,C^	4.6 ± 0.4 ^A^	24.7 ± 1.9 ^A^	4.3	6.4
Lentil	13.6 ± 0.6 ^B^	3.1 ± 0.3 ^A^	3.3 ± 0.2 ^B^	20.0 ± 0.7 ^C^	6.0	5.2
*p*-value	<0.001	<0.001	<0.001	<0.001		

^a^ Values are expressed as percent of dry weight; means with different superscripts within a column are significantly different (*p* < 0.05); ^b^ Means followed by different superscripted letters within a column are significantly different (*p* < 0.05) according to Tukey’s mean separation test; ^c^ Serving size is ½ cup of cooked, drained pulse; ^d^ This is per 100 kcal pulse crop as eaten. Abbreviations: IDF: insoluble dietary fiber; SDF: soluble dietary fiber; OLIGO: total oligosaccharides (raffinose + stachyose + verbascose); TDF: total dietary fiber (IDF + SDF + OLIGO).

**Table 6:** Range of dietary fiber content (%) among pulse crops.

Crop	IDF ^a^	SDF ^a^	OLIGO ^a^	TDF ^a^
Chickpea	14.4–17.1	2.0–5.8	1.0–3.5	19.5–24.9
Common bean	12.3–15.7	5.8–9.8	3.6–5.2	24.1–27.4
Dry pea	14.2–19.8	3.3–5.3	4.0–5.4	22.3–28.0
Lentil	12.3–14.7	2.7–3.9	3.0–3.7	18.4–21.3

^a^ Values are expressed as percent of dry weight. Abbreviations: IDF: insoluble dietary fiber; SDF: soluble dietary fiber; OLIGO: total oligosaccharides (raffinose + stachyose + verbascose); TDF: total dietary fiber (IDF + SDF + OLIGO).

**Table 7:** Dietary fiber, protein, and lipid content of pulse and cereal crops per 100 kcal edible portion.

Crop ^a^	Bean ^b^	Lentils ^c^	Chickpeas ^d^	Dry Pea ^e^	Wheat ^f^	Rice ^g^	Corn ^h^	Soy ^i^	Peanut ^j^
Protein g/100 kcal	6.4	7.8	5.4	6.4	4.9	2.3	3.6	8.9	4.3
Lipid g/100 kcal	0.5	3.3	1.6	0.3	1.4	0.8	1.6	4.5	6.9
Fiber g/100 kcal	6.6 ^k^	5.2 ^k^	5.3 ^k^	6.4 ^k^	0.8	1.3	2.5	3.0	2.8

^a^ Values for food composition taken from USDA Nutrient Database for Standard reference: Release 28; ^b^ Bean, pinto, mature seeds, cooked, boiled. Database reference number: 16043; ^c^ Lentils, mature seeds, cooked, boiled. Database reference number: 16070; ^d^ Chickpea, mature seeds, cooked, boiled. Database reference number: 16057; ^e^ Pea, green, cooked, boiled. Database reference number: 11305; ^f^ Bread, whole wheat, commercially prepared. Database reference number: 18075; ^g^ Rice, brown, long grain, cooked. Database reference number: 20037; ^h^ Corn, sweet, yellow, cooked, boiled. Database reference number: 11168; ^i^ Soybeans, green, cooked, boiled. Database reference number: 11451; ^j^ Peanuts, all types, cooked, boiled. Database reference number: 16088; ^k^ Values taken from Table 5.

## Abbreviations

The following abbreviations are used in this manuscript:

IDF	Insoluble dietary fiber
SDF	Soluble dietary fiber
OLIGO	Oligossacharide
TDF	Total dietary fiber

## References

[1] Otten J.J., Hellwig J.P., Meyers L.D. (2006). Dietary Reference Intakes.

[2] Mobley A.R., Jones J.M., Rodriguez J., Slavin J., Zelman K.M. (2014). Identifying practical solutions to meet America’s fiber needs: proceedings from the Food & Fiber Summit. Nutrients.

[3] Jones J.M. (2014). CODEX-aligned dietary fiber definitions help to bridge the ‘fiber gap’. Nutr. J..

[4] Thompson H.J., Brick M.A. (2016). Perspective: Closing the dietary fiber gap: An ancient solution for a 21st century problem. Adv. Nutr..

[5] Food and Agriculture Organization Definition and Classification of Commodities: Pulses and Derived Products. http://www.fao.org/es/faodef/fdef04e.htm.

[6] Gepts P., Famula T. (2012). Biodiversity in Agriculture: Domestication, Evolution, and Sustainability.

[7] Bressani R. (1983). Research needs to up-grade the nutritional quality of common beans (*Phaseolus vulgaris*). Plant Foods Hum. Nutr..

[8] Mitchell D.C., Lawrence F.R., Hartman T.J., Curran J.M. (2009). Consumption of dry beans, peas, and lentils could improve diet quality in the US population. J. Am. Diet. Assoc..

[9] Mudryj A.N., Yu N., Hartman T.J., Mitchell D.C., Lawrence F.R., Aukema H.M. (2012). Pulse consumption in Canadian adults influences nutrient intakes. Br. J. Nutr..

[10] Hipsley E.H. (1953). Dietary “fibre” and pregnancy toxaemia. Br. Med. J..

[11] Burkitt D.P., Walker A.R., Painter N.S. (1972). Effect of dietary fibre on stools and the transit-times, and its role in the causation of disease. Lancet.

[12] Trowell H. (1976). Definition of dietary fiber and hypotheses that it is a protective factor in certain diseases. Am. J. Clin. Nutr..

[13] Trowell H.C., Burkitt D.P. (1987). The development of the concept of dietary fibre. Mol. Aspects Med..

[14] Alimentarius CODEX (CODEX) Guidelines On Nutrition Labeling CAC/GL 2-1985. http://www.fao.org/ag/humannutrition/33309-01d4d1dd1abc825f0582d9e5a2eda4a74.pdf.

[15] McCleary B.V., DeVries J.W., Rader J.I., Cohen G., Prosky L., Mugford D.C., Champ M., Okuma K. (2010). Determination of total dietary fiber (CODEX definition) by enzymatic-gravimetric method and liquid chromatography: collaborative study. J. AOAC Int..

[16] McCleary B.V., DeVries J.W., Rader J.I., Cohen G., Prosky L., Mugford D.C., Okuma K. (2012). Determination of insoluble, soluble, and total dietary fiber (CODEX definition) by enzymatic-gravimetric method and liquid chromatography: collaborative study. J. AOAC Int..

[17] Kleintop A.E., Echeverria D., Brick L.A., Thompson H.J., Brick M.A. (2013). Adaptation of the AOAC 2011.25 integrated total dietary fiber assay to determine the dietary fiber and oligosaccharide content of dry edible beans. J. Agric. Food Chem..

[18] Cho S.S., Nelson A. (2012). Dietary Fiber and Health.

[19] Jones J.M. (2013). Dietary fiber future directions: integrating new definitions and findings to inform nutrition research and communication. Adv. Nutr..

[20] Cho S.S., Qi L., Fahey G.C., Klurfeld D.M. (2013). Consumption of cereal fiber, mixtures of whole grains and bran, and whole grains and risk reduction in type 2 diabetes, obesity, and cardiovascular disease. Am. J. Clin. Nutr..

[21] Park Y., Subar A.F., Hollenbeck A., Schatzkin A. (2011). Dietary fiber intake and mortality in the NIH-AARP diet and health study. Arch. Intern. Med..

[22] McRorie J.W. (2015). Evidence-based approach to fiber supplements and clinically meaningful health benefits, part 1: What to look for and how to recommend an effective fiber therapy. Nutr. Today.

[23] McRorie J.W. (2015). Evidence-based approach to fiber supplements and clinically meaningful health benefits, part 2: What to look for and how to recommend an effective fiber therapy. Nutr. Today.

[24] International Organization for Migration (IOM) Accelerating Progress in Obesity Prevention: Solving the Weight of the Nation. www.iom.edu/Reports/2012/Accelerating-Progressin-Obesity-Prevention.aspx.

[25] Tosh S.M., Yada S. (2010). Dietary fibres in pulse seeds and fractions: Characterization, functional attributes, and applications. Food Res. Int..

[26] Brummer Y., Kaviani M., Tosh S.M. (2015). Structural and functional characteristics of dietary fibre in beans, lentils, peas and chickpeas. Food Res. Int..

